# Effect of Vitamin-D on Glycemic Parameters and Adiponectin in gestational diabetes

**DOI:** 10.12669/pjms.40.8.9308

**Published:** 2024-09

**Authors:** Amna Nadeem, Ayesha Sadiqa, Muniza Saeed

**Affiliations:** 1 Amna Nadeem, M.Phil. Department of Physiology, CMH Lahore Medical College and Institute of Dentistry, Lahore, Pakistan. National University of Medical Sciences, Rawalpindi, Pakistan; 2 Ayesha Sadiqa, M.Phil. Ph.D. Department of Physiology, CMH Lahore Medical College and Institute of Dentistry, Lahore, Pakistan. National University of Medical Sciences, Rawalpindi, Pakistan; 3 Muniza Saeed, M.Phil. Department of Physiology, PGMI – Post Graduate Medical Institute, Lahore, Pakistan

**Keywords:** Blood glucose, Adiponectin, Gestational Diabetes, Pregnancy-Induced Diabetes, and Vitamin-D Deficiency

## Abstract

**Objective:**

To determine the effect of Vitamin-D-supplementation on glycemic parameters: glucose levels in blood, insulin, HbA1c, HOMA-IR, and adiponectin in women with gestational diabetes.

**Methods:**

An experimental study was executed at PGMI/LGH of Lahore from June 2020 to June 2021, with 34 Vitamin-D-deficient women who had gestational diabetes (20–26 weeks). All were aged between 21–32 years, randomly and equally divided into controls and cases. Cases received 200,000 IU Vitamin-D-dose. Fasting blood was collected before as well as after treatment from each participant. Spectrophotometry and peroxidase method were used to estimate HbA1c and glucose concentrations respectively. Insulin, adiponectin, and Vitamin-D were assessed by ELISA. To verify data normality, the Shapiro-Wilk test was applied and to prove group comparison Mann-Whitney U, Wilcoxon signed-rank, and Sample-t tests were used via IBM-SPSS version-21.

**Results:**

No difference in blood glucose was found between controls and cases before treatment (p=0.858), while post-treatment, significant reduction found in cases (p=0.019). Before treatment, no difference was noticed in insulin levels of both groups (p=0.44), however, post-treatment, a significant decline was expressed in cases (p=0.001). No difference was found in HOMA-IR between controls and cases before treatment (p=0.14) but post-treatment, significant reduction was observed in cases (p=0.001). Non-significant difference was noted in HbA1c before (p=0.664) and after (p=0.169) treatment in both groups. Non-significant upsurge in adiponectin was observed in cases before (p=0.544) and after (p=0.194) treatment.

**Conclusion:**

Vitamin-D supplementation significantly improves glycemic control in gestational diabetic women, however, its effect on adiponectin was non-significant.

## INTRODUCTION

Gestational Diabetes Mellitus (GDM) is a condition in which intolerance develops to glucose at first in gestation and remains in the later trimesters.^1^ During pregnancy the glucose uptake reduces by 40-60% along with marked insulin resistance i.e. three times compared to a non-pregnant state.^2^,^3^ To compensate for this, blood insulin is increased up to twice the normal levels, and if β-cells of Langerhans (pancreas) are unable to secrete the required insulin to encounter that raised glucose then GDM develops.^4^ The rate of GDM varies globally due to different diagnostic criteria. According to a meta-analysis in 2021, the worldwide GDM prevalence is 14%, based on the screening criteria developed by the International Association of Diabetes and Pregnancy Study Groups (IADPSG).^5^

Currently, the methods for estimating insulin resistance are the hyperinsulinemic-euglycemic clamp and the intravenous glucose tolerance test. Simple methods validated for deriving indices include homeostasis model assessment (HOMA), here HOMA-IR is taken by multiplying glucose levels (mg/dl) with insulin levels and dividing the value by 405.^6^ Vitamin-D (VD) plays an important role with low serum levels being linked to insulin sensitivity and β-cells efficacy.^7^ It maintains bone mineralization through calcium and phosphorus regulation. VD has also non-skeletal effects on the cardiovascular, endocrine, and immune systems.^8^

The prevalence of GDM in Pakistan is 9.47% in 2021.^9^ It is essential to control the incidence of GDM to prevent both neonatal as well as maternal complications. To our knowledge, no such study has been conducted in Pakistan on this topic. The objectives of this study were to determine the effect of vitamin-D supplementation on glycemic parameters: glucose levels in blood, insulin, HbA1c, HOMA-IR, and adiponectin in women with gestational diabetes.

## METHODS

An experimental study was conducted with 34 pregnant women within 20–26 weeks of gestation from June-2020 to June-2021, after receiving an ethical approval letter from the Advanced Studies and Research Board in their 96^th^ meeting. The letter no. was UHS/Education/126-14/3292 and the issuing date was December 22, 2014. All the subjects were aged between 21-32 years, were vitamin-D deficient, and had GDM. Those who smoked, had multiple gestations, or experienced other gestational issues, were excluded.

The study was accomplished following the Helsinki Declaration of Human Rights and was also ethically approved by the Ethical Committee of the Post Graduate Medical Institute (PGMI), at Lahore. All the participants belonged to the Gyne-Obs. Department of Lady Aitchison and Lahore General (LGH) Hospitals.

All participants were randomly divided into cases-*seventeen* and controls-*seventeen*. The case group received a single I/M dose of 200,000 IU of Vitamin-D (Indrop D-α) in addition to routine antenatal supplementation, while the control group was only given the routine antenatal vitamin supplements per oral. Medical history was taken from each participant, and afterward, every participant proceeded for a complete general physical and antenatal thorough examination. An oral glucose tolerance test (OGTT) was employed to confirm GDM and serum levels of Vitamin-D less than 29 in ng/ml were labeled as deficient for VD. A fasting blood sample of 5 ml was taken under aseptic conditions from each participant to assess blood glucose, serum insulin, HbA1c, adiponectin, and VD levels. Samples were taken before and 12 weeks after the intervention (Vitamin-D injection). ELISA technique was used to measure serum insulin (μIU/ml), adiponectin (μg/ml), and Vitamin-D (ng/ml).

The peroxidase method was used to estimate fasting blood glucose (mg/ml), while a spectrophotometer was used to measure HbA1c (%), and the Homeostatic Model Assessment of Insulin Resistance (HOMA-IR) was estimated as suggested by Singh and Saxena, 2010.^7^ The data was analyzed through IBM-SPSS version-21. To verify the normality of data, the Shapiro-Wilk test was employed. As blood glucose, HbA1c, adiponectin, and Vitamin-D estimations were normally distributed, so, an independent sample t-test, and for non-normal distributed data i.e. HOMA-IR and insulin levels, Mann-Whitney U and Wilcoxon signed-rank tests were applied respectively to compare the two groups. P≤0.05 was considered statistically significant.

## RESULTS

Two groups were compared: cases and controls, each with 17 women with GDM and VD deficiency. There was only a slight variance (p=0.054) in the average age (in years) between the cases (27.0±1.7) and controls (25.24±3.2). Before treatment, there was a negligible difference in the average BMI (kg/m²) between the cases and controls. The cases had an average BMI of 23.38±1.138, while the controls had an average BMI of 23.23±1.280 (p=0.726). Post-treatment, there was a noticeable difference in the BMI of the cases and controls. Specifically, the BMI of the cases was 27.74±1.331 while that of the controls was 29.30±1.879 (p=0.009).

Prior to treatment, the blood glucose level in controls was found to be 10.4% higher compared to post-treatment. Although, this difference was non-significant (p=0.617). The control group expressed a 20.3% reduction in serum insulin concentration before treatment than after treatment. This difference was highly significant (p=0.001). The control group exhibited a noteworthy 11.3% decrease in HOMA-IR levels before treatment compared to the post-treatment (p=0.030). Similarly, the same group revealed a decrease of 9.3% in HbA1c before treatment compared to after treatment. Also, this noticed difference was statistically significant (p=0.032). Before treatment, a minor decrease of 5.7% was found in serum VD levels than after treatment, though the statistical difference was noted as non-significant (p=0.437). The controls also expressed a significant increase of 69.2% in serum adiponectin levels before treatment than after treatment (p=0.001) (Table-I).

**Table-I:** Pre and post-treatment comparison of study parameters between comparative groups.

Study groups	Study Parameters	Pre-treatment		Post-treatment		P-value

		Mean±SD*	Median(IQR*)	Mean±SD*	Median(IQR*)	
Controls (n=17)	Blood glucose (mg/dl)	121.7±10.3	120(15)	110.2±15.1	112(27)	0.617
	Serum Insulin (µIU/ml)	18.5±6.9	19.3(12.60)	23.2±6.2	21.9(6.95)	0.001*
	HOMA-IR	5.5±1.9	5.48(2.84)	6.2±1.66	6.07(1.89)	0.030*
	HbA1c %	4.9±0.65	5.0(0.62)	5.4±1.1	5.3(1.05)	0.032*
	Vitamin-D (ng/ml)	16.03±4.3	18.2(7.75)	17.0±4.8	17.0(6.30)	0.437
	Serum Adiponectin (µg/ml)	3.4±1.51	2.9(5.89)	2.01±0.37	1.97(1.24)	0.001*
Cases (n=17)	Blood glucose (mg/dl)	122.3±10.6	115(18.5)	96.1±18.4	100(0.36)	0.001*
	Serum Insulin (µIU/ml)	13.5±3.3	12.3(4.2)	15.7±3.2	14.4(3.90)	0.003*
	HOMA-IR	4.0±0.9	3.74(0.96)	3.6±0.7	3.79(0.90)	0.102
	HbA1c %	5.0±0.6	5.0(0.95)	5.0± 0.6	5.0(0.55)	0.826
	Vitamin-D (ng/ml)	15.4±3.8	14.7(5.85)	33.9±6.2	35.1(9.9)	0.001*
	Serum Adiponectin (µg/ml)	3.7±1.66	3.2(5.53)	2.29±0.79	2.25(3.15)	0.001*

* SD = Standard Deviation, *IQR = Inter Quartile Range *HOMA-IR = Homeostatic Model Assessment of Insulin Resistance, *p≤0.05 is taken as significant.

Following the treatment, cases revealed decreased blood glucose levels by 27.3%, which was also proved as significant (p=0.001). Before treatment, the same cases also expressed a marked decrease in serum insulin levels by 14.0% compared to after treatment (p=0.003). Prior to treatment, a relatively low serum HOMA-IR level of 11.1% was observed in the cases, which was a non-significant difference compared to the post-treatment (p=0.102). Here, the same group expressed no significant difference in their HbA1c levels before and after the intervention (p=0.826). During pre-treatment, there was a significant decrease of 54.5% in serum VD levels was observed compared to post-treatment (p=0.001). During pre-treatment, the case group revealed a significant (p=0.001) decrease of 61.5% in serum adiponectin levels than during post-treatment, (Table-I).

During the pre-treatment, there was a minor rise of 0.5% in the blood glucose concentrations in cases compared to controls (p=0.858). Before treatment, statistically, a non-significant decline of 27.0% in serum insulin levels was found in cases compared to controls (p=0.44). The HOMA-IR decreased by 27.3% during pre-treatment in cases than in controls, though the difference was non-significant (p=0.14). Prior to treatment, a small increase of 2.04% in HbA1c levels was observed in cases than controls (p=0.664). A non-significant decline of 3.9% was observed before treatment in serum VD in cases than controls (p=0.511). Earlier to treatment, the cases revealed an increase of 8.8% in serum adiponectin than the control group, though the difference was non-significant (p=0.544) (Table-II).

**Table-II:** Statistical comparison of parameters between study groups before and after intervention.

Study groups	Study Parameters	Cases (n=17)		Controls (n=17)		P-value

		Mean±SD*	Median(IQR*)	Mean±SD*	Median(IQR*)	
Pre-treatment	Blood glucose (mg/dl)	122.3±10.6	115(18.5)	121.7±10.3	120(15)	0.858
	Serum Insulin (µIU/ml)	13.5±3.3	12.3(4.2)	18.5±6.9	19.3(12.60)	0.44
	HOMA-IR	4.0±0.9	3.74(0.96)	5.5±1.9	5.48(2.84)	0.14
	HbA1c %	5.0±0.6	5.0(0.95)	4.9±0.6 5	5.0(0.62)	0.664
	Vitamin-D (ng/ml)	15.4±3.8	14.7(5.85)	16.03±4.3	18.2(7.75)	0.511
	Serum Adiponectin (µg/ml)	3.7±1.66	3.2(5.53)	3.4±1.51	2.9(5.89)	0.544
Post-treatment	Blood glucose (mg/dl)	96.1±18.4	100(0.36)	110.2±15.1	112(27)	0.019*
	Serum Insulin (µIU/ml)	15.7±3.2	14.4(3.90)	23.2±6.2	21.9(6.95)	0.001*
	HOMA-IR	3.6±0.7	3.79(0.90)	6.2±1.6 6	6.07(1.89)	0.001*
	HbA1c %	5.0±0.6	5.0(0.55)	5.4±1.1	5.3(1.05)	0.169
	Vitamin-D (ng/ml)	33.9±6.2	35.1(9.9)	17.0±4.8	17.0(6.30)	0.001*
	Serum Adiponectin (µg/ml)	2.29±0.79	2.25(3.15)	2.01±0.37	1.97(1.24)	0 .194

* SD=Standard Deviation, *IQR= Inter Quartile Range *HOMA-IR=Homeostatic Model Assessment of Insulin Resistance, *p≤0.05 is taken as significant.

After treatment, the study cases showed a significant decrease in fasting blood glucose concentrations than controls (p=0.019). During post-treatment, the case group had a significant drop of 32.3% in serum insulin levels than controls (p=0.001). Following treatment, a noticeable difference between cases and controls. Cases had a 41.9% increase in HOMA-IR (p=0.001). Additionally, during post-treatment, cases had a significant increase of 99.4% in serum VD concentration compared to controls (p=0.001). Statistically, a non-significant upsurge of 13.9% during post-treatment was observed in serum adiponectin levels in the cases compared to controls (p=0.194) (Table-II).

## DISCUSSION

As present study expressed a significant difference between the controls and the interventional group for their insulin concentrations, where the controls exhibited a net increase of 4.7±3.6 μg/dl in insulin and the interventional group expressed a net rise of 2.2±2.7 μg/dl in insulin levels. These results are consistent with the findings of another study performed along the same lines with the administration of vitamin-D that reduces the raised biomarkers i.e. HbA1c and Fasting Blood Glucose in diabetic patients.^10^ Parallel to our results, a local study concluded that generally Pakistani pregnant women commonly expressed vitamin-D deficiency and this deficiency can be improved through maternal supplementation of vitamin-D.^11^ Another local study also declared that a significant positive correlation exists between vitamin-D in the mother and in the neonate.^12^

A significant relationship between low vitamin-D and increased risk of developing GDM.^13^ Quite similar to our findings, Ali et al. and Zahid et al. from Pakistan expressed their findings that pregnant women with GDM showed 71.80% higher frequency in vitamin-D deficiency compared to those pregnant who were not with GDM.^14^ Another study conducted on pregnant women with GDM found that maternal vitamin-D was significantly lower in such patients during mid-gestation. The same study reported that majority of such patients were vitamin D deficient.^15^ Here, in the same connection another study from Pakistan suggested the daily dose of 4000 IU vitamin-D supplementation in vitamin-D deficient pregnant women, the same study also declared that this dose is not only helpful in improving serum 25(OH)D levels in the gestational mothers but also improves 25(OH)D levels in their newborns.^16^

Another study from the local population concluded that in the Pakistani pregnant population, the deficiency of Vitamin-D is found very prevalent and this deficiency can be handled safely and effectively with a daily recommended dose of 5000 IU/day for pregnant women.^17^ However, the present study used a single experimental I/M dose of 200,000 IU of vitamin-D deficient pregnant women who also had GDM, in addition to their routine ante-natal supplementation, and found significant beneficial results with improved insulin levels in them.

An Indian study with the same study population found that 93% of GDM patients in their 24-28th week of gestation were also diagnosed with hypovitaminosis-D.^18^ Likewise another study reported that early pregnancy hypovitaminosis D is a considerable risk not only for GDM but also for its complications.^19^ Alongside, another study conducted on pregnant women with vitamin-D deficiency concluded that decreased vitamin-D may induce immune-inflammatory disorders in such patients and also declared an association between raised TSH and reduced vitamin-D, especially in the first trimester.^20^

Vitamin-D is known to enhance GLUT-4 expression in muscles, which then translocate in adipose tissue.^21^ Furthermore, vitamin-D also regulates insulin secretion by influencing both VDR and 1-α-hydroxylase, which is found in pancreatic β-cells that might be compromised in vitamin-D deficiency.^22^ As the present study observed that vitamin-D supplementation in vitamin-D deficient pregnant women with GDM not only increases insulin levels, and regulates glycemic index, but also lowers the serum adiponectin, another study from Pakistan conducted on the same study patients declared the significance of vitamin-D concentration monitoring in GDM patients with the concern that in such patients, an inverse association was noticed between 25-OH-D, parathyroid hormone, and bone mineral density.^23^ A similar conclusion was reported by another local study that via vitamin-D supplementation, the health of the maternal as well as neonatal body can be improved by in turn improving their bone mineral density. The same study revealed that GDM patients have a higher risk of vitamin-D deficiency during pregnancy and vitamin-D deficiency may affect bone turnover in GDM patients.^24^

### Limitation

Our study was limited by the fact that markers were not observed in the pre-gestational or post-partum period. In addition, the inclusion criteria specified a gestational age of 20-26 and the results of this study may not apply to other age groups.

## CONCLUSION

In conclusion, Vitamin-D is effective in controlling gestational diabetes, and further exploration and rigorous investigation into the mechanisms underlying the therapeutic effects of Vitamin-D holds promise for refining our understanding of its clinical implications and potentially unveiling novel avenues for effective interventions in GDM management. For women with gestational diabetes who are deficient in Vitamin-D, Vitamin-D supplementation can improve their glycemic control and boost their insulin levels. This supplementation can also lower their serum adiponectin concentrations.

### Authors’ Contribution:

**AN:** Designed, did data collection, statistical analysis & manuscript writing, is responsible for integrity of research.

**AS:** Conception, design, manuscript writing and revised it critically and manuscript writing.

**AS and MS:** Did data collection, review, and final approval of the manuscript.
